# mapPat: tracking pathogens evolution in space and time

**DOI:** 10.1093/bioadv/vbaf015

**Published:** 2025-02-07

**Authors:** Erika Ferrandi, Graziano Pesole, Matteo Chiara

**Affiliations:** Institute of Biomembranes, Bioenergetics and Molecular Biotechnologies, Consiglio Nazionale delle Ricerche, Bari 70126, Italy; Department of Biosciences, University of Milan, Milan 20133, Italy; Institute of Biomembranes, Bioenergetics and Molecular Biotechnologies, Consiglio Nazionale delle Ricerche, Bari 70126, Italy; Department of Biosciences, Biotechnology and Biopharmaceutics, University of Bari "A. Moro", Bari 70126, Italy; Department of Biosciences, University of Milan, Milan 20133, Italy

## Abstract

**Motivation:**

The COVID-19 pandemic highlighted the importance of genomic surveillance for monitoring pathogens evolution, mitigating the spread of infectious disorders, and informing decision-making by public health authorities. Since the need for the summarization and interpretation of large bodies of data, computational methods are critical for the implementation of effective genomic surveillance strategies.

**Results:**

Here, we introduce mapPat, an R Shiny application for the interactive visualization of pathogens genomic data in space and time. mapPat is designed as a user-friendly dashboard and allows the dynamic monitoring of the evolution of variants, lineages, and mutations in the genome of a pathogen at glance through informative geographic maps and elegant data visuals. mapPat provides a fine-grained map of pathogens evolution and circulation and represents a useful addition to the catalogue of bioinformatics methods for the genomic surveillance of pathogens.

**Availability and implementation:**

mapPat is available at GitHub (https://github.com/F3rika/mapPat.git).

## 1 Introduction

Genomic surveillance is a highly effective tool for monitoring the spread of pathogens and infectious disorders and informing decision-making in healthcare. However, the need for the analysis and interpretation of large bodies of data poses a considerable challenge.

The recent experience with the COVID-19 pandemic represented a turning point for the application of pathogens genomic surveillance in public health. Data and insights generated by large-scale sequencing and comparative analyses of SARS-CoV-2 genomes guided the development of effective prevention, containment, and control measures, including vaccines and molecular diagnostics. Moreover, genomic surveillance allowed the prompt identification and characterization of novel SARS-CoV-2 variants associated with potential epidemiological implications (Chiara *et al.* 2021).

Many novel bioinformatics tools were developed to assist in the analysis of COVID-19 data. The interpretation of the results and conclusions, however, were controversial at times, also due to the lack of clear, high-quality methods for the effective graphical representation and summarization of the data (Carter *et al.* 2022).

These considerations prompted the development of mapPat, an R Shiny (Chang *et al.* 2024) application for the interactive visualization of the distribution of viral variants, lineages, and mutations through time and across geographic locales.

## 2 Methods

### 2.1 Interface design

mapPat was developed in R-v4.4.1 under the Shiny-v1.9.1 framework. The following packages were used:

- Ggplot2-v3.5.1 (Wickham 2016): stacked area charts, barplots, and scatterplots.
- Pheatmap-v1.0.12 (Kolde 2019): heatmaps.
- Leaflet-v2.2.2 (Cheng *et al.* 2024a): choropleth maps.
- Rgeoboundaries-v1.3 (Dicko *et al.* 2024): geometric data for outlining political and administrative boundaries of regional territories defined according to the geoBoundaries API (Runfola *et al.* 2020).
- Htmltools-v0.5.8.1 (Cheng *et al.* 2024b): labels.
- RColorBrewer-v1.1-3 (Neuwirth 2022): colour palettes.

### 2.2 Input

mapPat collects data in a simple tabular format by processing publicly available metadata tables and associated sequences, either from GISAID (Khare *et al.* 2021) or NextStrain (Hadfield *et al.* 2018). These data are subsequently processed through HaploCoV (Chiara *et al.* 2023) and a collection of ancillary scripts (see Supplementary materials section 1). A collection of precomputed tables can be downloaded from a dedicated Zenodo Repository (https://doi.org/10.5281/zenodo.14163899). Additionally, mapPat also provides a built-in option that allows the user to select and download (if needed) datasets to be visualized.

Custom Python3 utilities are used to homogenize inconsistent annotation of geographic metadata when required. Only geographic names consistent with administrative levels as encoded by Rgeoboundaries (Dicko *et al.* 2024) and according to ISO-3 standard country codes are considered for data analysis (see Supplementary materials section 2 for more details).

## 3 Results

mapPat empowers genomic surveillance by enabling the dynamic tracking of viral lineages, genomic mutations, and variants, both at national and regional geographic level, through easily interpretable data visuals and informative maps and provides a set of unique features in the rich ecosystem of bioinformatics tools for the genomic surveillance of pathogens. For example, compared with phylogenetic-based methods, like Cluster-Tracker (McBroome *et al.* 2022), Taxonium (Sanderson 2022), or Treenome Browser (Kramer *et al.* 2023), mapPat offers a more rich set of features and data analytics and allows the direct comparison of multiple properties of a pathogen of interest at the same time. Moreover, unlike specialized methods such as CoV-Spectrum (Chen *et al.* 2022), mapPat is not limited to a specific pathogen and could ideally accommodate data from different human pathogens and microbes.

In mapPat information concerning variants, lineages and mutations can be explored through three distinct but interconnected tabs that provide informative visualizations with different levels of granularity. A brief overview of functionalities of mapPat is provided in the following sections.

### 3.1 Layout and user interface

The layout of mapPat is inspired by the past experience with SARS-CoV-2 genomic surveillance. Three tabs are used to display key features at different levels of granularity:

- **Variants Tab**: overview of the spread and distribution of the main variants of a pathogen as defined by international health authorities. This panel provides a broad representation of the circulation of variants and families of variants under scrutiny by health authorities.
- **Lineages Tab**: overview of the distribution of lineages, as defined by a nomenclature system, at national and regional level. Lineages represent distinct subgroups or clades within a named variant (see above). Hence, the analysis of lineages allows monitoring of specific viral clades with a higher level of granularity.
- **Mutations Tab**: breakdown of the novel mutations that accumulate in the viral genome and of changes in their frequency. mapPat defines the mutations that are characteristic of every lineage (as defined in a reference nomenclature, see above) based on available data. A mutation is considered characteristic if present in ≥50% of the genomes assigned to the lineage. Mutations that are not characteristic of a lineage, but show a relative frequency ≥1% at a specific locale for more than a week, are reported in this panel. The aim is to facilitate the identification of newly emerging mutations, lineages, or sub-lineages.

Users interact with mapPat by setting a series of filters and criteria through the control panel at the bottom of the user interface (see Supplementary materials section 4 for a detailed description).

First, a dataset, pathogen, country, and interval of time are selected. Time intervals are represented in the form of weeks (SARS-CoV-2) or months (other pathogens) with respect to an arbitrary date that normally corresponds with the day of first isolation/sequencing of the pathogen genome or with a significant epidemiological event (see Supplementary materials section 4).

**Figure 1.**
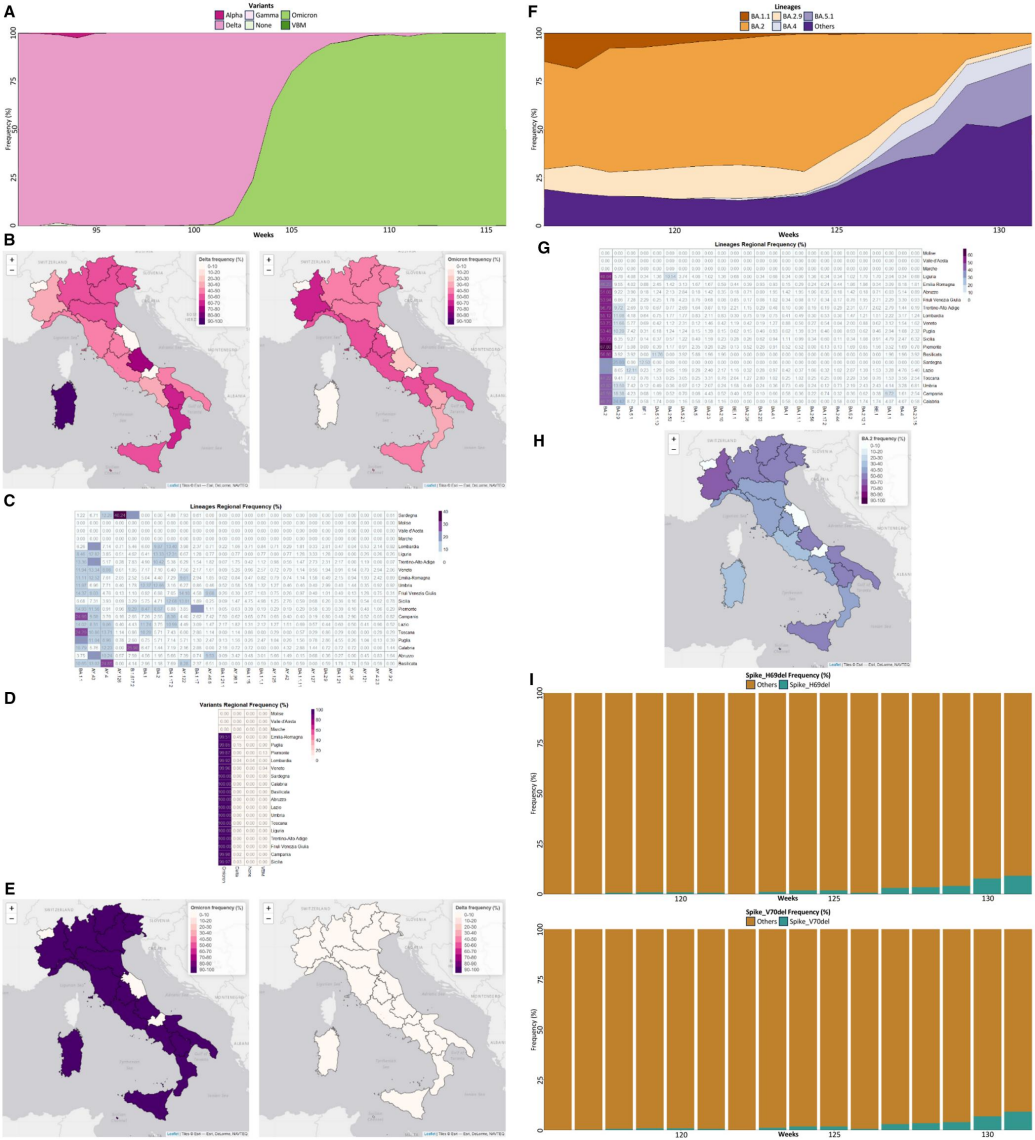
mapPat application and results. Application of mapPat for the study of SARS-CoV-2 variants of concern in Italy. A–C: weeks 91–116. D–I: weeks 116–131. A: Delta was the most prevalent variant in Italy up until around Week 105 and was completely replaced by Omicron by Week 116. B: The first Omicron isolates were identified in the centre-north Italian regions, while Delta was mostly spread in southern regions and islands. C: SARS-CoV-2 lineages presented an uneven distribution at regional level with the most frequent lineage being BA.1.1. D and E: Omicron replaced Delta in almost all Italian regions. F: The most prevalent Omicron lineages in Italy shifted from BA.1 and BA.2 to the newly identified BA.4 and BA.5 lineages. G and H: At regional level, BA.2 was the most circulating lineage, while other Omicron lineages presented a more localized propagation. I: Non-defining mutations H69del and V70del of the BA.2 lineage showed a sharp increase in frequency.

Once a selection is applied, data can be visualized and inspected. The number of available data points is summarized in a barplot (i.e. number of sequenced genomes), while stacked area charts are used to illustrate the relative prevalence of lineages and variants (Fig. 1A and F).

Specialized data visuals are used to display specific aspects of pathogen genome evolution. For example, the Variants Tab features a barplot with the breakdown of the five most prevalent lineages associated with a user-selected variant to facilitate the identification of lineages that are increasing and/or decreasing in prevalence. Similarly, the Mutations Tab illustrates the prevalence of the two most frequent non-defining mutations for a user-selected lineage of interest to highlight novel emerging mutations that accumulate in the genome (Fig. 1I). Only lineages showing a minimum prevalence above a user-selected threshold are displayed.

Throughout the 3 panels, heatmaps and choropleth maps are used to depict the local circulation of variants, lineages, and mutations and their regional frequencies in order to aid users in the visual identification of potentially relevant patterns, both at the national and regional levels (from Fig. 1B–E, G and H).

### 3.2 Application to a real use case

To showcase the application of mapPat, the tool was applied for a retrospective analysis of the spread and changes in prevalence of SARS-CoV-2 variants of concern in Italy. Two intervals of time were scrutinized: the first (from weeks 91 to 116) corresponds with the introduction of the Omicron variant in the country, and the second with the evolution and spread of the Omicron BA.2 lineage (from weeks 116 to 131). According to available data, Delta was the most prevalent SARS-CoV-2 variant in Italy between weeks 91 and 100, when genome sequences associated with the Omicron variant were first reported. By Week 116, Omicron completely replaced Delta and became the most prevalent variant in Italy (Fig. 1A). Interestingly, the first Omicron isolates were mostly associated with the centre-north regions, with a peak in frequency in Piedmont (Fig. 1B). On the other hand, Delta’s prevalence remained high in Sardinia, Abruzzo, and in general in the southern regions of the peninsula. The observed distribution of SARS-CoV-2 lineages at the regional level was uneven, with different lineages showing high levels of prevalence in different regions (Fig. 1C). The most prevalent Omicron lineage was BA.1.1, while AY.43 and AY.4 represented the most widespread lineages of the Delta variant. However, these lineages displayed a patchy distribution across different regions (Fig. 1C). By Week 131, Omicron completely replaced Delta in almost every Italian region (Fig. 1D and E); a shift in the most prevalent lineage from BA.1 to BA.2 (and derivatives) was also observed (Week 116). Subsequently, around Week 124, two new Omicron lineages, BA.4 and BA.5, started to circulate in Italy (Fig. 1F). Similar trends, characterized by a primary circulation of BA.2 and a more locally restricted circulation of other Omicron lineages, were also observed across different Italian regions (BA.5.1 in Lazio, BF.1 in Sardinia, BA.1.1 in Campania, among others, Fig. 1G and H). Interestingly, a sharp increase in the frequency of the non-defining mutations H69del and V70del was also observed in the same interval of time (Fig. 1I) in the BA.2 lineage. These mutations were previously linked with immune escape in SARS-CoV-2 (Harvey *et al.* 2021).

## 4 Conclusions

In the aftermath of the COVID-19 pandemic, the development of novel methods and approaches for tracking and monitoring the spread of human pathogens and their evolution remains a critical aspect for the design and implementation of containment measures by health authorities and to inform decision-making. Here, we introduce mapPat, a completely customizable and flexible tool for the interactive visualization of pathogens genomic data in time and at different levels of geographic granularity. Empowered with a rich collection of data visuals and geographic maps, mapPat can greatly facilitate the visual inspection of genomic surveillance data and the identification of potential epidemiologically relevant patterns. Although the tool was designed based on the previous experience with the COVID-19 pandemic, mapPat is of general application and can be easily adapted to investigate patterns of circulation and evolution of any microbe for which genomic data and structured geographic metadata are available. An example of the application of mapPat to the mPox virus is reported in the Supplementary materials section 5.

All in all, we believe that the unique set of features implemented by mapPat allows a comprehensive and easy-to-interpret summarization of pathogens genomic data and that our tool represents a valuable addition to pre-existing methods for pathogens genomic surveillance. Importantly, since the potential for accommodating data from any microorganism, the range of application of mapPat is not limited exclusively to pathogen genomic surveillance, and the method could be applied to study the phylogeographic patterns of evolution in any species/organism at glance.

## Supplementary Material

vbaf015_Supplementary_Data

## Data Availability

The data underlying this article are available in *Zenodo* at https://doi.org/10.5281/zenodo.14163899 and can be accessed with DOI: 10.5281/zenodo.14163900 (Version v1).
