# Supplementary material for: mapPat: tracking pathogens evolution in space and time
Source: Bioinform Adv. 2025 Feb 7;5(1):vbaf015. doi: 10.1093/bioadv/vbaf015 (PMC11835230; doi:10.1093/bioadv/vbaf015)
Supplement: vbaf015_Supplementary_Data [file vbaf015_supplementary_data.zip › mapPat_SupplementaryFormatted_FINAL.docx]

**Supplementary materials**

# **Input tables**

mapPat stores data in a collection of tables in simple text format that summarise key information about pathogens circulation and evolution at national and regional level.

These tables are derived by processing publicly available data downloaded from databases of viral genome sequences, such as NextStrain (Hadfield *et al.*, 2018) or GISAID (Khare *et al.*, 2021), through HaploCoV (Chiara *et al.*, 2023) and a collection of Perl and Python3 scripts.

All these scripts are all publicly available at GitHub (https://github.com/F3rika/mapPat/tree/mapPat_Current/InputGeneration) and can be used to convert large tabular files with metadata, such as the *metadata.tsv* files made available by GISAID or NextStrain, to the collection of tables used by mapPat.

When metadata files include precomputed lists of mutations for every entry, such as the metadata tables available for SARS-CoV-2 from either GISAID or Nextstrain, genomic sequences in FASTA format are not required. Conversely, genomic sequences in FASTA format need to be supplied for mPox and other pathogens for which precomputed sets of mutations are not available. In this case, HaploCoV is used to compute genomic variants with respect to a reference genome for all the entries.

The output consists in a large table in .tsv format with a summary of the metadata and the list of the variants for every isolate/genome sequence (also referred as HaploCoV format table). Tables in HaploCoV format are used as the main input to all the custom scripts.

A collection of precomputed tables, obtained by processing publicly available data, can be downloaded from a dedicated Zenodo repository (https://doi.org/10.5281/zenodo.14163899). These datasets can be selected through a dedicated drop-down menu and loaded natively by mapPat (detailed instructions are illustrated in the following sections).

Only countries with more than 1000 distinct available genome sequences for a pathogen of choice are included in mapPat and visualised through the application. A total of five tables (Counts tables, see below) are computed for every country that meets this minimum requirement. Six additional tables (Configuration tables, see below) are used to summarise available data and set the configuration of the tool’s widgets and tabs.

- **Counts tables**

Counts tables summarise the number of genomes associated with a specific variant, lineage or mutation per unit of time (weeks or months). Counts are recorded at both national and regional level. The preferred time unit (weeks or months) for counts tables depends on the amount of available data: currently weeks are used for SARS-CoV-2 and months for all the other pathogens, for example mPox.

Counts tables are space delimited and have a common layout. Units of time are represented in the columns, and data points for either variants, lineages or mutations are recorded in the rows. When required, counts tables also include additional columns to store metadata required to correctly group and represent different layers of granularity (i.e. regions or locales names, lineages names and similar).

Counts tables include:

1. ***Epiweek.Var.COUNTRY.csv***: number of sequenced genomes for every named variant (see below) at national level, per week/month.
2. ***Epiweek.COUNTRY.csv***: number of sequenced genomes for every named lineage at national level, per week/month.
3. ***HeatmapRegLin_COUNTRY.csv***: number of sequenced genomes for every named lineage at regional level, per week/month.
4. ***COUNTRY_muts_perLin.csv***: list of non-defining mutations (characterised by frequency >=1% and <50% in a specific country or locale for more than a week) and the number of sequenced genomes that present them, for every named lineage, per week/month at both national and regional level.
5. ***Total_COUNTRY_regions.csv***: total number of sequenced genomes calculated per week/month at regional level.

*COUNTRY* is a placeholder for countries ISO-3 codes (see below).

- **Configuration tables**
  Configuration tables summarise available data and information for every country included in mapPat and are used to set-up and specify the correct configuration of widgets and plots. These tables are tab delimited, with the features of interest represented by the columns and their values reported in the rows. Some configuration tables are used to set the general layout of mapPat (general), while others describe data available for a specific pathogen (pathogen specific).

Configuration tables include:

1. ***mapPat_inTabUpdates_Availability.txt*** (general): list of datasets that are available for visualisation using mapPat; associated file name and URL for downloading the latest datasets if needed are also reported.
2. ***PathogenSelection_ConfigTab.txt*** (general): list of pathogens for which counts tables are available; associated country defaults (ISO-3 code, see following section), time unit defaults and reference date for the computation of time offsets calculation are also reported.
3. ***countriesListTracker.txt*** (pathogen specific): complete list of ISO-3 country codes (see below) for all the countries with available data.
4. ***InTab_avCheck.txt*** (pathogen specific): associates each ISO-3 country code (see below) with the corresponding list of counts tables. In this table columns represent input files categories and rows represent countries. When available, file names are reported, otherwise NA (Not Available) is indicated.
5. ***CountryISOADM_AssocTab.txt*** (general): specifies the preferred administrative level (ADM according to RGeoboundaries) used to draw maps for every country. Also includes the corresponding country name and ISO-3 code (see following section).
6. ***LinVar_ConvTabTracker.txt*** (for SARS-CoV-2 only): associates lineages with the corresponding variants and classification status (VOC, VOI, VBM, further explanation below) according to the WHO classification guidelines (if any). Note that starting from March 16th 2023, since the Omicron variant became the only variant of SARS-CoV-2 circulating worldwide, the World Health Organisation (WHO) revised its nomenclature, centering it around Omicron and its lineages (further information can be found in WHO official statement at https://www.who.int/news/item/16-03-2023-statement-on-the-update-of-who-s-working-definitions-and-tracking-system-for-sars-cov-2-variants-of-concern-and-variants-of-interest).
   To accommodate for this revision of the nomenclature, *LinVar_ConvTabTracker.txt* contains a column to specifically indicate, only for lineages of the Omicron variant, those that could potentially impact Public Health (as per WHO guidelines) and are thus labelled as Variants Being Monitored (VBM).

# **Curation of geographic metadata annotation**

Geographic locales and administrative regions in mapPat are defined according to specifications from Rgeoboundaries (Dicko *et al.*, 2024), the R client for the geoBoundaries Database (Runfola *et al.*, 2020), a reference resource which provides information about administrative boundaries for almost every country in the world. In geoBoundaries countries are identified by their ISO-3 code, while regions or administrative districts by their extended names. ISO-3 are internationally recognised three letter codes used to identify countries and their subdivision in a way that allows to avoid the ambiguity and errors carried by names (which can change depending on language).

Unfortunately, many discrepancies in geographic data annotation were observed between databases of viral genome sequences and the ISO-3 and names standard used by mapPat and Rgeoboundaries. For example, in the case of SARS-CoV-2, only 64.17% of the data as provided in the GISAID (Khare *et al.*, 2021) database could be directly imported in the correct format in mapPat. Specifically, 8.03% of the total number of records in GISAID presented incomplete geographic metadata (missing regions), while for 27.81% of them region names could not be directly mapped to the names used by Rgeoboundaries due to both typos and inconsistent methods of annotation of geographic metadata (*Supplementary Figure 1-A*).

The *mapPat_CouRegAssTabGen_WF.py* Python3 script (available at https://github.com/F3rika/mapPat/tree/mapPat_Current/GeoAssociationGeneration) was developed to mitigate discrepancies in geographic metadata annotation and match countries and regions names from a database of choice (GISAID in the case of SARS-CoV-2) with the corresponding values as annotated in Rgeoboundaries. The algorithm implemented by this script is briefly described below.

All the substrings of length 3 of geographic names as included in Rgeoboundaries are indexed in a hash table. Subsequently, for every input name from external databases, a list of candidate matching names in Rgeoboundaries is built by searching in the hash table all the geographic names with at least a matching substring.

Finally, the Levenshtein Edit Distance (LED) is computed for every candidate match and the candidate with the lowest edit distance is considered the best match. Only best matches with LED<=3 are retained.

If both country and region names are available this procedure is repeated iteratively: first to find the best candidate or corresponding country name and subsequently to identify the best matching region name among all the regions included in the country.

To minimise data loss, both the administrative tiers of Rgeoboundaries, ADM1 (province, state or governorate) and ADM2 (district, municipality or commune), are considered.

As illustrated in *Supplementary Figure 1-B* using this procedure it was possible to match about 76.65% of the global data available for SARS-CoV-2 in the GISAID database to ISO-3 codes or geographic names used by Rgeoboundaries.

Notwithstanding this improvement, we noticed that for some countries, including the United States of America (USA), the procedure described above still failed to match geographic names of the country. To overcome the issue, country names were manually corrected to match the requirements of Rgeoboundaries.

*Supplementary Table 1* reports the complete list of countries that required this step of manual curation: for each country both the original and manually curated name are reported.

This process allowed to increase the total proportion of “matching” data to 83.53% (*Supplementary Figure 1-C*).

| **Original Name** | **Curated Name** |
| --- | --- |
| USA | United States |
| Central African Republic | Central African Rep |
| Czech Republic | Czechia |
| Democratic Republic of the Congo | Dem Rep of the Congo |
| Marshall Islands | Marshall Is |
| Republic of the Congo | Rep of the Congo |
| Saint Kitts and Nevis | St Kitts & Nevis |
| Saint Vincent and the Grenadines | St Vincent & the Grenadines |
| Solomon Islands | Solomon Is |

***Supplementary Table 1*. List of original and curated names for countries requiring manual curation.** Original Name: countries as annotated in GISAID; Curated Name country names as referenced by Rgeoboundaries.

Similar inconsistencies to those observed in reporting the names of the countries were recorded for the names of the geographic regions and annotation of regional data. A further round of manual curation was performed to resolve these additional inconsistencies. In this case only the first eleven countries with the highest number of lost data entries (corresponding to a span of about 30%-100% data loss due to mismatches in regions names per country) were considered. The complete list is reported in *Supplementary Table 2*.

A significant proportion of the entries reported an incorrect administrative level in the database (i.e. cities instead of regions or districts). Belgium and Slovakia were excluded from manual curation since the requirement for a substantial amount of work, which lies outside the scope of this work.

This process allowed to further increase the proportion of data available to mapPat to 90.71% (*Supplementary Figure 1-D*).

|  | **BEFORE CURATION** | | | | |  | **AFTER CURATION** | | | | |
| --- | --- | --- | --- | --- | --- | --- | --- | --- | --- | --- | --- |
| **Country** | **#All** | **#Mat** | **#Unm** | **%Mat** | **%Unm** |  | **#All** | **#Mat** | **#Unm** | **%Mat** | **%Unm** |
| Germany | 945558 | 396502 | 528219 | 41.93 | 55.86 |  | 945558 | 924721 | 0 | 97.80 | 0.00 |
| Belgium | 182294 | 35291 | 145504 | 19.36 | 79.82 |  | 182294 | 35291 | 145504 | 19.36 | 79.82 |
| Spain | 252007 | 130593 | 121231 | 51.82 | 48.11 |  | 252007 | 251581 | 243 | 99.83 | 0.10 |
| Ireland | 111048 | 181 | 110601 | 0.16 | 99.60 |  | 111048 | 110782 | 0 | 99.76 | 0.00 |
| Sweden | 274182 | 162634 | 110572 | 59.32 | 40.33 |  | 274182 | 273045 | 161 | 99.59 | 0.06 |
| Poland | 95056 | 3549 | 86905 | 3.73 | 91.43 |  | 95056 | 90103 | 351 | 94.79 | 0.37 |
| Austria | 274851 | 183419 | 81977 | 66.73 | 29.83 |  | 274851 | 265382 | 14 | 96.55 | 0.01 |
| Czechia | 61424 | 9659 | 48937 | 15.73 | 79.67 |  | 61424 | 56662 | 1934 | 92.25 | 3.15 |
| Slovakia | 47494 | 0 | 47354 | 0.00 | 99.71 |  | 47494 | 0 | 47354 | 0.00 | 99.71 |
| Lithuania | 43120 | 158 | 42762 | 0.37 | 99.17 |  | 43120 | 42420 | 500 | 98.38 | 1.16 |
| Slovenia | 87455 | 0 | 41987 | 0.00 | 48.01 |  | 87455 | 41987 | 0 | 48.01 | 0.00 |

***Supplementary Table 2*. Available data entries before and after region names manual curation.** This table reports the complete list of countries for which manual curation of region names was performed. A detailed breakdown of data that could be imported in mapPat before and after the process is reported. #All: total number of data entries. #Mat: total number of matched entries in mapPat. #Unm: total number of unmatched entries in mapPat. %Mat: % of matched annotations (wrt total number of entries). %Unm: % of unmatched annotations (wrt total number of entries).

In summary, by combining manual curation and our custom algorithm for the matching of geographic names, data loss due to inconsistent reporting of geographic data was reduced from 27.81% (*Supplementary Figure 1-A*) to 3.65% (*Supplementary Figure 1-D*).

Complete data counts and frequencies are available through Supplementary Data.


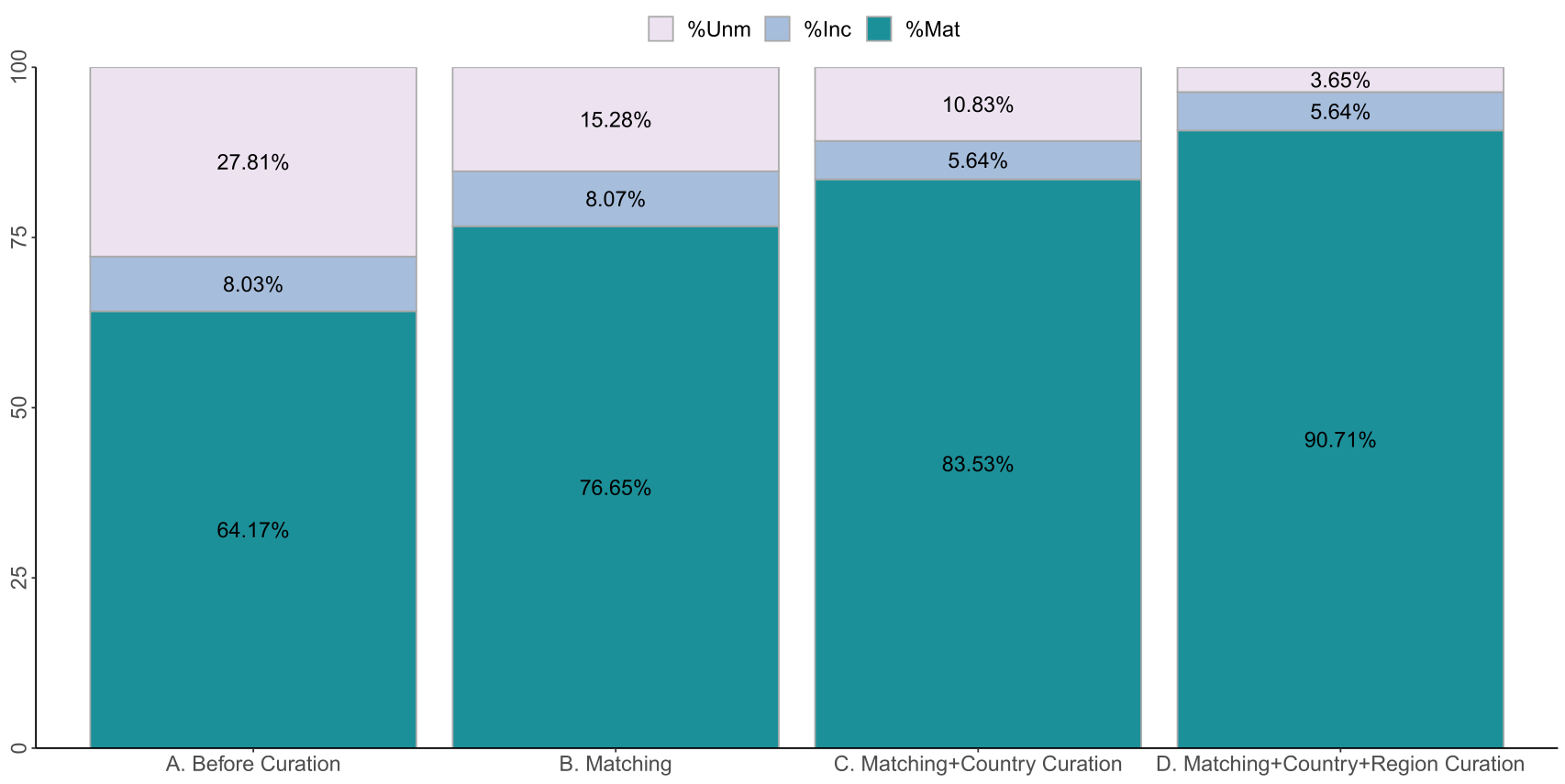


***Supplementary Figure 1*. Percentage (%) of matched data entries between GISAID and mapPat through different rounds of data curation.** Percentage (%) of matched data (%Mat), data with incomplete annotation (%Inc) and unmatched data (%Unm) through different levels of data curation. ***A*.** No data curation. ***B*.** Automatic matching of names by custom script. ***C*.** Custom script and manual curation of country names. ***D*.** Custom script, manual curation of country names and manual curation of region names

# **Global and relative frequencies and their usage in mapPat**

mapPat represents pathogens circulation by showing changes in the global or relative frequency of variants, lineages and mutations. Methods used to compute frequencies and their applications are detailed below:

- **Global frequency**

Global frequency is computed as the ratio between the total number of genomes associated with a variant, lineage or mutation and the total number of available genomes. It can be calculated both at national and regional level.

Global frequency is used in heatmaps and choropleth maps to describe variants and lineages distribution at regional level. Furthermore, it can also be used for filtering data of interest through specific widgets (see following sections for further explanations).

- **Pointwise frequency**

Represents the frequency of a variant, lineage or mutation at a single time point (week or month). It is calculated as the ratio between the number of genomes associated to a variant, lineage or mutation and the total number of genomes sequenced nationally at that week/month.

Pointwise frequency is used in area charts to highlight variations in variants and lineages distribution at national level along the user-selected time period of interest.

- **Relative frequency (global or pointwise)**

Represents the global or pointwise frequency of a lineage or mutation relative to a selected variant or lineage. For the computation of relative frequencies only data associated with the selected variant or lineage is considered.

Relative frequency is mainly used in the Variants and Mutations Tabs to describe the relative prevalence of lineages and mutations with respect to a variant or lineage of choice. It can also be used as a filtering tool.

More specifically, relative pointwise frequency is represented through barplots to facilitate the tracking of changes in the prevalence of lineages associated with a variant of interest, or to highlight variations in the prevalence of non-defining mutations in a user-selected lineage.

Relative global frequency is used in heatmaps and choropleth maps to display the distribution of mutations at different geographic locales.

# **Filters and data selection**

In mapPath users operate data selection by setting thresholds and criteria through the widgets at the bottom of the user interface. Selections are applied dynamically. Some conditions are general and are applied to data visualised throughout all the tabs, while some are specific to only a single tab.

- **General configuration widgets**

The following settings are common to all tabs and govern the first layer of data selection. This group of widgets allows the customization of selected data throughout all the tabs and includes:

- ***Dataset*** (drop down menu and action button): selection of the dataset to be displayed. Only one dataset can be selected at a time. If needed, datasets are automatically downloaded from the dedicated Zenodo Repository (https://doi.org/10.5281/zenodo.14163899). Note that the selected dataset is loaded to mapPat only after confirming the selection through the “Load dataset” action button.
- ***Pathogen*** (drop down menu): selection of the pathogen to be displayed. Only one pathogen can be selected at a time.
- ***Country*** (drop down menu): selection of countries. The list of available countries depends on the user-selected pathogen (only countries with more than 1000 available genomes are included).
- ***Time range*** (slider): specification of the interval of time of interest. In mapPat time points are computed in the form of offsets (in weeks or months) with respect to a fixed date, which is set (arbitrarily) for each pathogen in the *PathogenSelection_ConfigTab.txt* configuration file. SARS-CoV-2: 2019-12-30. mPox: 2022-01-01.

MapPat is composed of 3 tabs that serve different purposes.

The Variants and Lineages Tabs provide an accurate snapshot of the circulation of specific “viral types” and of changes in their prevalence across countries and regions at set time intervals.

The Mutations Tab aims to facilitate the monitoring of the evolution of viral lineages by tracking novel mutations as they accumulate in the genome through time, at both national and regional/local level.

The categorization of viral pathogens used by mapPat adheres to the system developed by the World Health Organization (WHO) and by the scientific community for the nomenclature and classification of SARS-CoV-2 throughout the COVID-19 pandemic.

Lineages represent monophyletic groups or clades of closely related viral isolates, characterised by a common set of mutations, while higher order groups of lineages with a common evolutionary origin (common ancestor) associated with novel epidemiological characteristics or traits (such as increased infectivity, disease severity, immune escape and more) are called variants.

According to WHO, variants can be classified in up to three distinct classes:

1. **Variants Being Monitored (VBM)**: suspected to have a growth advantage with respect to other circulating variants. Currently used only to classify Omicron sub-variants (see previous sections).
2. **Variants Of Interest (VOI)**: verified growth advantage with respect to other circulating variants, increasing prevalence and number of cases over time (indicating the possibility of an emerging risk for global public health).
3. **Variants Of Concern (VOC)**: variant meeting the definition of VOI and associated with an observable impact on disease severity and/or changes in epidemiology.

Dedicated widgets are used to apply custom selections in specific tabs.

- **Tab specific configuration parameters**

The complete list of tab specific widgets, organised by tab, is available below:

- Variants Tab widgets
- ***Category*** (drop down menu): class of variants to be displayed. The available options are All, VOC, VOI, VBM and None. This classification includes all the variants that were labelled with a greek alphabet letter by WHO at any time throughout the COVID-19 pandemic. The VBM category allows to align this nomenclature with the current World Health Organisation (WHO) guidelines for SARS-CoV-2 variants classification (see previous sections for further information).
- ***Variant*** (drop down menu): specific variant to be analysed. The prevalence of the circulating lineages associated with the selected variant is displayed in the form of a barplot. Only variants with at least one sequenced genome in the time interval selected by the user can be chosen.
- ***Min % of genomes*** (radio buttons): used to set the minimum relative global frequency (%, calculated at national level) required to include a named lineage associated with the user-selected variant for graphical representation. The following presets are available: 1%, 2.5%, 5%, 7.5%, 10%. Lineages that do not meet the minimum prevalence threshold are aggregated under “Others”.
- ***Variant (Map 1)*** and ***Variant (Map 2)*** (drop down menus): selection of up to 2 variants for which regional global frequency (%) is shown through choropleth maps. Only variants with at least one sequenced genome in the time interval set by the user can be displayed.
- Lineages Tab widgets
- ***Min % of genomes*** (radio buttons): used to set the minimum global frequency (%, calculated at national level) required to consider a lineage for graphical representation. The following presets are available: 1%, 2.5%, 5%, 7.5%, 10%. Lineages that do not meet the minimum prevalence threshold are aggregated under “Others”.
- ***Number of lineages*** (drop down menu): maximum number *N* of lineages that can be explicitly represented in plots. Only the top *N* most prevalent lineages will be displayed. The available options are integers from 1 to 10. Lineages that are not explicitly represented are aggregated under “Others”.
- ***Lineage (Map 1)*** and ***Lineage (Map 2)*** (drop down menus): selection of up to 2 lineages for which regional global frequency (%) is shown through choropleth maps. Only lineages with a global prevalence (%) at national level above the selected threshold (see above) in the time interval of interest can be displayed.
- Mutations Tab widgets
- ***Lineage*** (drop down menu): lineage for which non-defining mutations are displayed in the Mutations Tab. Only lineages with a global prevalence (%) at national level above the selected threshold (see list above) in the time period of interest can be selected.
- ***Mutation (Map 1)*** and ***Mutation (Map 2)*** (drop down menus): selection of up to 2 mutations for which relative regional global frequency (%) is shown through choropleth maps. Only mutations with a relative global prevalence (%) at national level above 1% in the time interval of interest can be selected.

# **mapPat for the study of mPox in the United States**

To demonstrate the applicability of mapPat for tracking the circulation of any pathogen, the tool was used to inspect the spread and change in prevalence of mPox lineages in the United States of America.

The interval of time selected for this analysis spanned from month 5 to month 25 (see previous sections for how time is calculated in mapPat) and covered the most part of the 2022 mPox outbreak in the countries where this virus is non-endemic. The severity of the clinical manifestation of the disease and the lack of links to countries where the circulation of mPox is endemic prompted WHO to declare it a Public Health Emergency of International Concern (PHEIC) on July 23rd 2022 (further information is available at WHO official website through dedicated pages at https://www.who.int/news-room/fact-sheets/detail/monkeypox and https://www.who.int/emergencies/situations/monkeypox-oubreak-2022).

Data was loaded in mapPat and inspected visually. Time units were expressed in months, time intervals were computed starting from 2022-01-01 and the analysed time period included months from 5 to 25.

According to available data the first genomes of mPox in the USA were reported at month 5, while the peak of circulation (1257 genomes sequenced in total) was observed at month 14 (*Supplementary Figure 2-A*). The most prevalent lineages all belonged to Clade II (Hadfield *et al.*, 2018), with B.1, B.1.2 and B.1.20 being the most frequent ones along the whole time interval of interest (*Supplementary Figure 2-A*). A similar trend was observed also at regional level in different states. B.1 and B.1.2 were the most prevalent lineages globally and displayed a 100% frequency in South Dakota (B.1), New Hampshire (B.1) and West Virginia (B.1.2) (*Supplementary Figure 2-B* and *Supplementary Figure 2-C*). Interestingly, regional data highlighted the presence of two additional lineages with local circulation B.1.7 and B.1.13, which were the most prevalent in Idaho (B.1.7), Vermont (B.1.7) and Kentucky (B.1.13) respectively (*Supplementary Figure 2-B*). The non-defining mutations 170262_G|A and 178133_G|A were observed in the B.1 lineage both at national and local level (*Supplementary Figure 2-D* and *Supplementary Figure 2-E*). Both variants had a patchy distribution at regional level (*Supplementary Figure 2-F*).

***
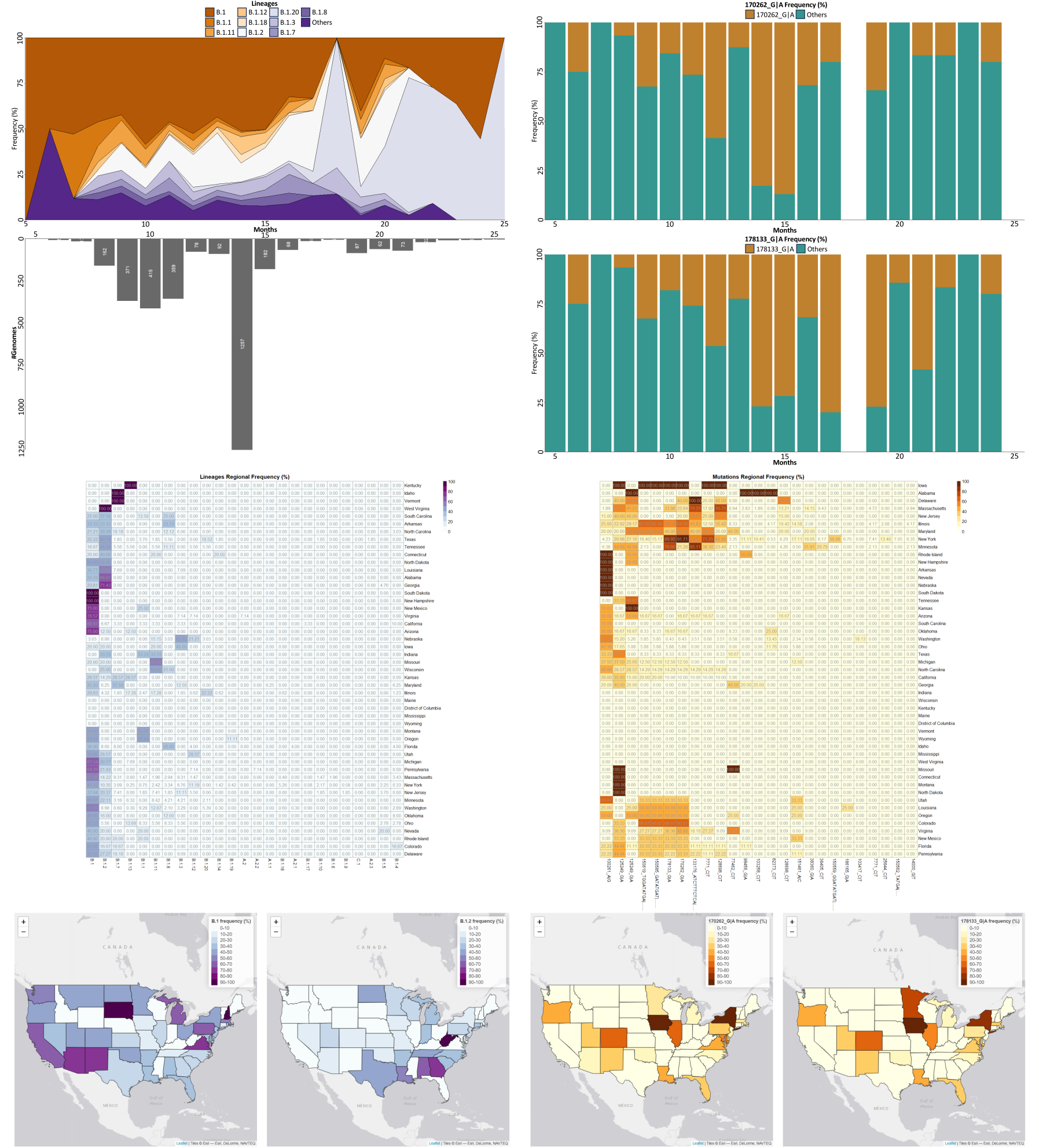
***

***Supplementary Figure 2*. Application of mapPat to mPox.** Visualisation of mPox genome sequencing data in the United States during the 2022 outbreak. Data from month 5 to month 25 are represented. ***A*.** mPox started spreading around month 5 with a peak at month 14. The most widespread lineages were B.1, B.1.2 and B.1.20 (Clade II). ***B-C*.** B.1 was the most circulating lineage in South Dakota and New Hampshire, while B.1.2 reached 100% frequency in West Virginia. Notably, B.1.7 and B.1.13 presented a lower circulation nationally, but local data showed that they were prevalent in Idaho, Vermont and Kentucky respectively. ***D-F*.** The non-defining mutations 170262_G|A and 178133_G|A were observed in the B.1 lineage both nationally and locally (higher circulation in specific states).

References

Brister,J.R. *et al.* (2015) NCBI Viral Genomes Resource. *Nucleic Acids Res.*, **43**, D571–D577.

Chiara,M. *et al.* (2023) HaploCoV: unsupervised classification and rapid detection of novel emerging variants of SARS-CoV-2. *Commun. Biol.*, **6**, 1–15.

Dicko,A. *et al.* (2024) rgeoboundaries: geoBoundaries Client. R package version 1.3. *https://CRAN.R-project.org/package=rgeoboundaries.*

Hadfield,J. *et al.* (2018) Nextstrain: real-time tracking of pathogen evolution. *Bioinformatics*, **34**, 4121–4123.

Khare,S. *et al.* (2021) GISAID’s Role in Pandemic Response. *China CDC Wkly.*, **3**, 1049–1051.

Runfola,D. *et al.* (2020) geoBoundaries: A global database of political administrative boundaries. *PLOS ONE*, **15**, e0231866.
